# Maslinic Acid Inhibits the Growth of Malignant Gliomas by Inducing Apoptosis via MAPK Signaling

**DOI:** 10.1155/2022/3347235

**Published:** 2022-06-28

**Authors:** Yongqiang Wang, Hewei Zhang, Zusen Ye, Qiang Ye, Xuezhi Yang, Wei Mao, Ruoting Xu, Yanlei Zhang

**Affiliations:** ^1^Department of Surgery, Key Laboratory of Diagnosis and Treatment of Severe Hepato-Pancreatic Diseases of Zhejiang Province, Zhejiang Provincial Top Key Discipline in Surgery, The First Affiliated Hospital of Wenzhou Medical University, Wenzhou, Zhejiang, China; ^2^Department of Neurology, The First Affiliated Hospital of Wenzhou Medical University, Wenzhou, Zhejiang, China; ^3^Hospital of Traditional Chinese Medicine of Zhenhai, Ningbo, Zhejiang, China

## Abstract

**Background:**

Gliomas are primary malignant brain tumors. Despite recent advances in surgery and clinical neuro-oncology, the prognosis of patients with glioma is still poor. Therefore, there is an urgent need to find new therapeutic drugs.

**Methods:**

Here, we have studied the anticancer effect of maslinic acid in glioma and explored its potential molecular mechanism. CCK-8, Ki67 immunofluorescence, and colony formation tests are used to detect the proliferation of glioma cells. Transwell and migration experiments are used to detect the function of cell invasion and migration, and RNA-seq was performed to identify differentially expressed genes. Western blot analysis helps us identify important signaling pathways. Finally, the anticancer effect of maslinic acid was confirmed in vivo through tumor xenografting experiments.

**Results:**

Our experiments obtained high-throughput data on the treatment of maslinic acid in glioma. We found that maslinic acid significantly inhibits the proliferation, invasion, and migration of glioma cells and promotes the apoptosis of glioma cells via suppressing MAPK signaling.

**Conclusions:**

This is the first time to analyze the mechanism of maslinic acid against glioma based on transcription. Our experiments show that maslinic acid may be a useful natural product for the treatment of glioma.

## 1. Introduction

Gliomas are the most frequent tumors of the central nervous system and include diffuse and circumscribed gliomas. [1]. Malignant gliomas account for approximately 60% of primary brain tumors in adults [2]. In recent years, the prognosis of patients with malignant glioma has not changed significantly [3]. Despite cytoreductive surgery, radiotherapy, and cytotoxic chemotherapy, the median survival time is 9–12 months, and few patients are cured from this disease [2]. Postoperative chemotherapy is the normal treatment for gliomas, and most malignant gliomas are resistant to chemotherapy drugs [4]. Therefore, it is necessary to develop new drugs for the chemotherapy treatment of patients with malignant glioma.

Maslinic acid is a triterpenoid which was the natural compound widely distributed in medicinal plants [5]. Recent studies have shown that maslinic acid has strong anticancer activity. It has been identified that it can play anticolon cancer [6], lung cancer [7], breast cancer [8], pancreatic cancer [9], kidney cancer [10], and prostate cancer effects by inhibiting cell proliferation and promoting cell apoptosis [11, 12]. Research by Martin et al. showed that maslinic acid can affect the growth and survival of glioma cell lines by regulating the accumulation of reactive oxygen species, but the specific mechanism has not been elucidated [13].

Mitogen-activated protein kinase (MAPK) is a family of serine/threonine kinases that play an important role in linking cell surface receptors to changes in transcription programs. MAPK is part of a three-component kinase module consisting of MAPK, upstream MEK, and MEKK. MEKK couples signals from cell surface receptors to trigger downstream pathways. A large number of studies have shown that these proteins play a key role in the regulation of a variety of cellular processes, including cell migration, proliferation, differentiation, and apoptosis [14, 15].

This study found that maslinic acid inhibited the proliferation, migration, and invasion of malignant glioma cells and induced their apoptosis. In vivo, maslinic acid can inhibit the growth of transplanted tumors in nude mice. In order to study the mechanism of action, we first used a high-throughput sequencing method to comprehensively analyze the mechanism of action of maslinic acid. Our research indicates that maslinic acid may be a potential therapeutic drug for malignant glioma.

## 2. Materials and Methods

### 2.1. Cell Lines

Human glioma cell lines U87 and U251 were obtained from American Type Culture Collection (ATCC, Manassas, VA, USA) and maintained in DMEM cell culture medium (Gibco, Grand Island, NY, USA) supplemented with 10% FBS (Gibco, Grand Island, NY, USA) and 1% ampicillin and streptomycin (Gibco) in the incubator at 37°C with 5% CO_2_.

### 2.2. CCK-8 Assay

According to the manufacturer's instructions, the Cell Counting Kit-8 (CCK-8) cell proliferation and cytotoxicity test kit was used to measure the viability of U87 and U251 cells that had been treated with different concentrations of maslinic acid. First, the 96-well plates, each well containing 100 *μ*l cell suspension (5 × 10^3^ cells), was placed in the 37°C incubator for 24 h and treated with the different concentrations of maslinic acid. After that, 10 *μ*l of the reagent at different concentrations was added to each pore and incubated for an additional 2 h. In comparison with untreated control cells, the differences in these experimental cells' viability were observed at 450 nm by a Multiskan Spectrum spectrophotometer. All the experiments were done in the same way for 3 times.

### 2.3. Colony Formation Assay

The U87 and U251 cells were inoculated in a 6-well plate with 1000–2000 cells/well. The 30 *μ*M and 60 *μ*M of maslinic acid was mixed as soon as the cells grew into colonies that could be observed by the naked eyes. Following 24 h of treatment, the cell colonies were counted after being fixed with formaldehyde and stained with crystal violet.

### 2.4. Wound-Healing Assay

The U87 and U251 cells were inoculated in a 6-well plate with 200000 cells/well and incubated at 37°C for 2 days. Next, a linear gap was scratched in the confluent cell monolayer in the culture area with a crystal pipette tip. Then, the deciduous cells were washed away with PBS and the medium with 30 *μ*M and 60 *μ*M of maslinic acid was added. Subsequently, the cells gradually filled the gap and the images of the culture area were observed by an inverted microscope every 24 h.

### 2.5. Transwell Assay

The transwell assay was used to assess the invasion capacities of U87 and U251 cells in vitro. Maslinic acid was added to the upper chamber containing 500 *μ*l serum-free medium of cell suspension (5 × 10^3^ cells). The cells were coated with Matrigel® reduced by the growth factor for the invasion test, and in order to act as a chemical inducer, 10% FBS was added to the lower chamber. In the following 24 hours, formaldehyde and 0.5% crystal violet were used to stain the invaded cells that migrated through the membrane and attached to the membrane's bottom. The final result of invaded cells was shown in five randomly selected fields under a microscope.

### 2.6. Immunofluorescence Assay

The U87 and U251 cover slip cells that had been cultured with 30 *μ*M and 60 *μ*M of maslinic acid in 6-well plates were fixed in 4% formaldehyde for 20 min after washing with phosphate-buffered saline (PBS) once for 1 min and permeabilized with 0.1% Triton X-100 for 15 min. Then, the cells were blocked with 2% BSA in 1X PBS for 30 min at 37°C. Next, the primary antibody of Ki67 (1 : 1000) was used to stain the fixed cells overnight at 4°C. After that, they were incubated with appropriate secondary antibodies at 37°C for 1 h. At last, the cells were stained by DAPI nuclear for 3 min and presented with a fluorescence microscope.

### 2.7. RNA-Seq Analysis

The data set included six U251 cell samples, treated with maslinic acid (0 *μ*M and 30 *μ*M) for 24 h in three biological replicates. Total RNA was extracted from cells and purified using TRIzol reagent (Invitrogen), and shipped to LC-Bio Technology company (Hangzhou, China). RNA-seq library and RNA-seq were constructed by Illumina Novaseq™ 6000 and used for further RNA-SEQ detection and analysis. Differentially expressed mRNAs were selected according to fold change >2 or fold change <0.5 and *p*-value <0.05 by R package edgeR or DESeq2 [16, 17]. The differentially expressed genes (DEGs) were subjected to Gene Ontology (GO) enrichment and Kyoto Encyclopedia of Genes and Genomes (KEGG) enrichment analysis.

### 2.8. Western Blotting

U87 and U251 human malignant glioblastoma cell lines were seeded in a 6-well plate and treated with different concentrations of maslinic acid for 24 h. Total proteins were isolated from U87 and U251 cells. Protein concentrations were determined by a BCA protein assay kit (Beyotime Biotechnology, Shanghai, China). Total proteins (30 *μ*g) from each sample were separated in SDS-PAGE and transferred to an activated PVDF membrane (IPVH00010, Millipore, Massachusetts, USA). After that, proteins were blocked with 5% skim milk in TBST for 1°h at room temperature and then incubated overnight at 4°C with a primary antibody. After three washes (10 min each) in TBST, membranes were incubated with the secondary antibodies for a further 1 h at room temperature. The protein bands were visualized via chemiluminescence detection on autoradiographic film. Proteins were quantified by measuring the intensity of signals using Image-Pro Plus and normalized to that for the GAPDH antibody. The primary antibodies used in this study include anti-Cleaved Caspase-3 (1 : 1000, Abcam, ab32042), anti-BCL-2 (1 : 1000, Abcam, ab265331), anti-BAX (1 : 1000, Abcam, ab133557), anti-P-ERK (1 : 1000, Proteintech, 25654-1-AP), and anti-GAPDH (1 : 1000, Cell Signaling Technology, 5174S) antibodies.

### 2.9. Immunohistochemical Analysis

Immunohistochemical analysis was performed using 4 *μ*m thick sections that had been dewaxed with xylene and rehydrated in sequential ethanol. Sections were incubated in 0.1% sodium citrate buffer (pH 6.0) for antigen retrieval, and endogenous peroxidase activity was blocked with 3% hydrogen peroxide (Beyotime, Shanghai, China). IHC staining was performed using the following primary antibodies: anti-PCNA (1 : 200). Stained sections were examined and images were taken using a DM4000B LED Microscope System (Leica Microsystems) and a DFC 420C 5 M Digital Microscope Camera (Leica Microsystems). The integrated optical density (IOD) was measured using Image-Pro Plus software (version 6.0).

### 2.10. In Vivo Tumor Xenograft Study

The experimental mice (BALB/c), male, 5–7 weeks old, and weighing 18−23 g, were received from the Experimental Animal Centre of Wenzhou Medical University (Wenzhou, China). Mice lived in a comfortable environment under suitable temperature and light. Meanwhile, the mice were fed standard feed and water regularly. The experimental mice were randomly divided into two groups. Each group was subcutaneously injected with 5 × 10^6^ U251 cells in 10 *μ*l of PBS, respectively. And both the groups received a subcutaneous injection of control solvent (0.9% physiological saline) and the appropriate concentrations of maslinic acid (20 mg/kg·d), respectively, for 6 days. The tumor volume was calculated every 3 days. In addition, it should be noted that the longest one was selected regardless of length or width, based on formula *V* = (length × width^2^)/2.

### 2.11. Histopathological Analysis

The tumor specimens from experimental mice (BALB/c) were fixed in formalin and embedded in paraffin. Then they were cut into 5 *μ*m sections and stained with hematoxylin and eosin. At last, the final images were captured by DM4000B LED microscope system and a DFC420C5M digital microscope.

### 2.12. Statistical Analysis

Data were presented as the mean ± standard error of the mean (SEM). All statistical analyses were performed using GraphPad Prism software (version 8.0, GraphPad Software). Two-sided Student's *t*-test was used to analyze the differences between the two groups. A one-way analysis of variance was used when more than two groups were compared. A *P* value less than 0.05 was considered statistically significant.

## 3. Result

### 3.1. Maslinic Acid Inhibits Proliferation of Glioma Cells

**Figure 1:**
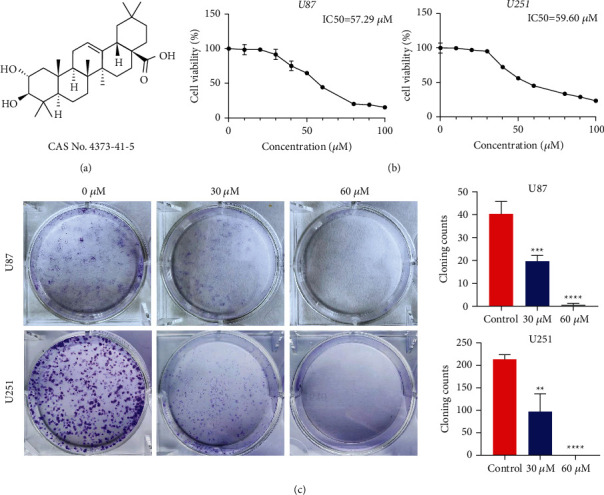
Maslinic acid-induced inhibition of the proliferation of U87 and U251 cells. (a) The chemical structure of maslinic acid. (b) CCK-8 Analysis of U87 and U251 cells incubated with maslinic acid (0, 30, 60 *μ*M). (c) The effects of maslinic acid on the colony formation of U87 and U251. The cells were exposed to maslinic acid (0, 30, 60 *μ*M) for 24 h After 14 days cells were stained with crystal violet and colony counted. Data are representative of three independent experiments, expressed as mean ± SD. ^*∗∗*^*p* < 0.01, ^*∗∗∗*^*p* < 0.001, and ^*∗∗∗∗*^*p* < 0.0001.

**Figure 2:**
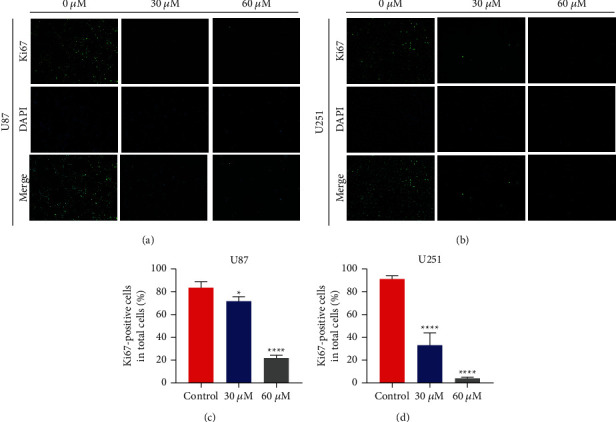
Maslinic acid-induced inhibition of the expression of ki67 protein of human glioma cells. Ki67 Immunofluorescence following U87 (a) and U251 (b) cells incubation with maslinic acid (0, 30, and 60 *μ*M) for 24 h. Scale bar = 50 *μ*m. (c) Quantitative analysis of Ki67-positive cells in U87 cells. (d) Quantitative analysis of Ki67-positive cells in U251 cells. Data are representative of three independent experiments and expressed as mean ± SD. ^*∗*^*p* < 0.005 and ^*∗∗∗∗*^*p* < 0.0001.

Following the addition of the maslinic acid, the different performances of U87 and U251 cells were shown by CCK-8, colony formation assay, and Ki67 immunofluorescence and analysis. It was shown in Figure 1(b) that the activity of U87 and U251 cells did decrease with the increase of drug concentration. Meanwhile, the number of U87 and U251 cells with 30 *μ*M and 60 *μ*M maslinic acid was considerably lower than the control group (Figure 1(c)). Therefore, the colony formation experiments also proved that U87 and U251 cells proliferation was significantly inhibited at the concentrations of 30 *μ*M and 60 *μ*M of maslinic acid. At last, it was as expected that the result of the decreased cell proliferation with maslinic acid at 30 *μ*M (Figure 2(a)) and 60 *μ*M (Figure 2(b)) was captured by Ki67 immunofluorescence and analyses compared with the untreated group (Figures 2(c) and 2(d)). However, maslinic acid of 30 *μ*m did not affect the proliferation of Human Kidney-2 (HK-2) cells, Normal Human Pancreatic Duct Epithelial (HPDE) cells, and Human Umbilical Vein Endothelial Cells (HUVEC) (Figure S1). All of the above experiments proved that the growth of these cells could be inhibited with the maslinic acid at the appropriate concentration.

### 3.2. Maslinic Acid Inhibits Migration and Invasion of Glioma Cells

**Figure 3:**
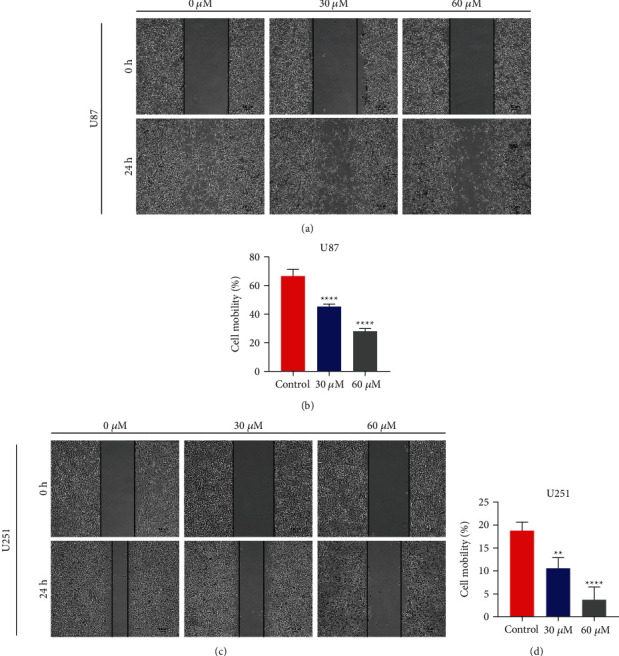
Maslinic acid-mediated inhibition of the migration of U87 and U251 cells. Wound-healing assay analysis of U87 (a) and U251 (c) cells incubated with maslinic acid (0, 30, and 60 *μ*M) for 24 h. (b) The quantification results of migration U87 cell number. (d) The quantification results of the U251 cell number. Data are representative of three independent experiments and expressed as mean ± SD. ^*∗∗*^*p* < 0.01 and ^*∗∗∗∗*^*p* < 0.0001. Scale bar = 100 *μ*m.

**Figure 4:**
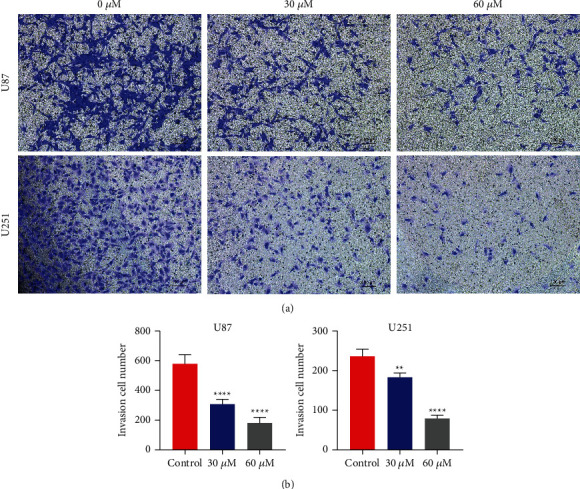
Maslinic acid-mediated inhibition of the invasion of human glioma cells. (a) Transwell assay following U87 and U251 cells incubated with maslinic acid (0, 30, and 60 *μ*M) for 24 h demonstrating that maslinic acid significantly inhibited invasion of U87 and U251 cells of glioma in a concentration-dependent manner. (b) The quantification results of invasion cell number. Data are representative of three independent experiments and expressed as mean ± SD. ^*∗∗*^*p* < 0.01 and ^*∗∗∗∗*^*p* < 0.0001. Scale bar = 100 *μ*m.

And then, after treatment of U87 (Figures 3(a) and 3(b)) and U251 (Figures 3(c) and 3(d)) cells with maslinic acid, there was a greater reduction in cell migration by the wound-healing experiments. Moreover, U87 and U251 cells added with DMSO did show more exaggerated migration capability before being treated with the maslinic acid (Figures 4(a) and 4(b)). In summary, maslinic acid could obviously inhibit glioma cell invasion and migration.

### 3.3. Enrichment Analysis of GO and KEGG Pathways in Differently Expressed Genes

**Figure 5:**
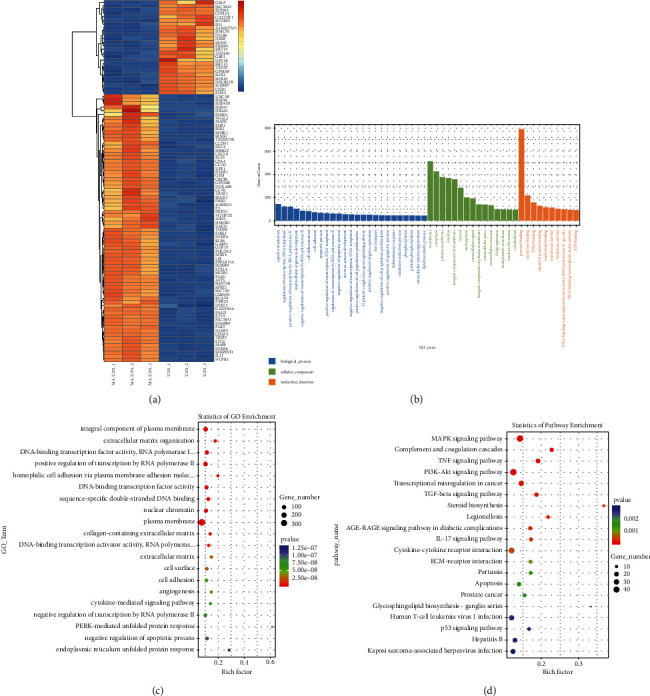
GO and KEGG pathway enrichment analysis of DEGs between U251 and maslinic acid-treated U251. (a) The cluster heat map of the first 100 DEGs in the U251 and maslinic acid-treated U251 datasets. The abscissa indicates the number of samples, whereas the ordinate indicates DEGs. The histogram in the upper right corner represents the color level; each rectangle corresponds to the expression value of a sample. (b) GO term of Top 25, Top 15, and Top 10. According to the number of differential genes annotated to GO Term, they were arranged in descending order, showing the distribution of the number of significantly different genes in GO Term enriched in biological processes, cell components, and molecular functions. (c, d) GO enrichment analysis and KEGG pathway enrichment analysis of DEGs in U251 cells post maslinic acid treatment. The results showed the GO Term and pathway of the top 20 enriched significantly in the form of a scatter plot.

The total RNA of U251 samples that treated with maslinic acid (0 *μ*M and 30 *μ*M) for 24 h were extracted and analyzed by RNA-Seq. On the basis of the similarity of gene expression profiles in different samples, different genes were clustered to show the expression of different genes in U87 and U251 cells treated with maslinic acid. Different levels of gene expression were shown in different colors in a heat map, which presented the top 100 genes with the smallest *p*  value. To elaborate a little further, the abscissa is the sample, and the ordinate is the screened differently expressed genes. As shown in Figure 5(a), the range from blue to white to red indicates the expression level from low to high. Blue is an expression of genes that are under expressed; meanwhile, red means a high expression of genes (Figure 5(a)).

According to the GO enrichment analysis, there is a significant correlation of the DEGs in the maslinic acid group, among integral components of the plasma membrane, extracellular matrix organization, DNA-binding transcription factor activity, and RNA polymerase II-specific (Figure 5(c)). Similarly, the statistics of pathway enrichment also expressed a great connection in the maslinic acid group with the MAPK signaling pathway, Complement and coagulation cascades, and TNF signaling pathway (Figure 5(d)). At last, the DEGs were classified according to the biological process (BP), cell composition (CC), and molecular function (MF). In the biological process group, DEGs were mainly enriched in signal transduction, regulation of transduction, DNA-templated, and positive regulation of transduction by RNA polymerase II. In the aspect of cellular components, the DEGs were focused on membrane, cytoplasm, and plasma membrane. The DEGs were mainly concentrated in protein binding, metal ion binding, and DNA binding for molecular function (Figure 5(b)).

### 3.4. Induction of Cancer Cell Apoptosis by Maslinic Acid Depends on the MAPK and Caspase-3/Bcl-2/Bax Apoptotic Signaling Pathways

**Figure 6:**
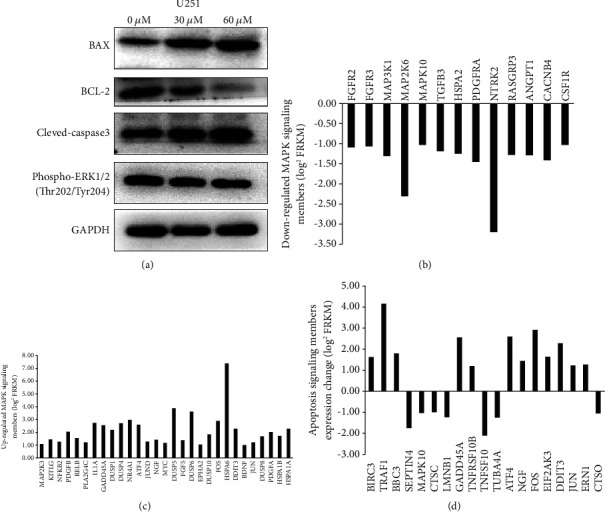
Maslinic acid induces U251 cell apoptosis. (a) Western blot analysis showed the changes in the MAPK signal pathway and apoptosis-related proteins in U87 and U251 cells. Maslinic acid inhibits the MAPK signal pathway which promotes the apoptosis of tumor cells and exerts its antitumor effect. (b) Downregulated MAPK signaling members (log2 FRKM). (c) Upregulated MAPK signaling members (log2 FRKM). (d) Apoptosis signaling members' expression change (log2 FRKM).

Analysis of the KEGG pathway showed that the MAPK signaling pathway was the most significantly upregulated signaling pathway (Figure 5(d)). Among the genes related to the MAPK signaling pathway, 29 genes were downregulated (Figure 6(b)) and 13 genes were upregulated (Figure 6(c)). We further verified the sequencing results with the western blot method, and the western blot results showed that maslinic acid treatment enhanced the expression of p-ERK1/2 (Figure 6(a)). In addition, it is noteworthy that maslinic acid treatment significantly regulated the expression of apoptosis genes, and the expression of 19 apoptosis-related genes was changed. Western blot results showed that maslinic acid upregulated the expression of proapoptotic family members cleaved-caspase-3 and Bax but decreased the expression of antiapoptotic protein BCL-2 (Figure 6(a)).

### 3.5. Maslinic Acid Inhibits Tumor Growth of Glioma Cells in Nude Mice

**Figure 7:**
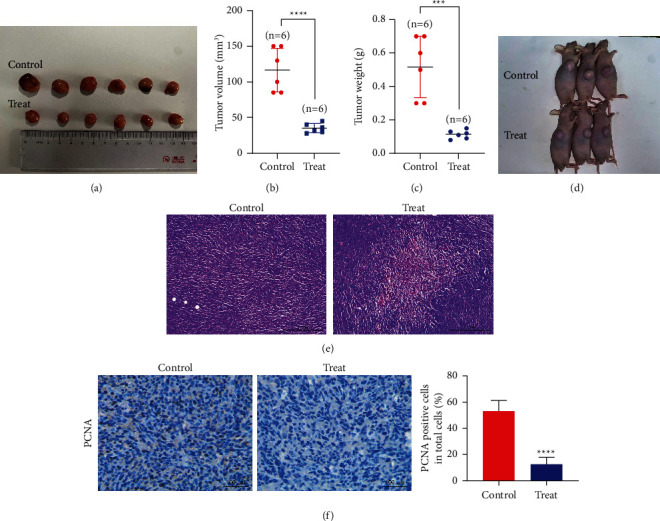
Maslinic acid inhibits the growth of U251 xenografts in nude mice. (a–d) U251 cells were inoculated subcutaneously into nude mice to establish a xenograft tumor model. After the successful establishment of the nude mouse model, the nude mice were randomly allocated into two groups and injected intraperitoneally with PBS or maslinic acid, respectively. After 14 days of treatment, the tumor size and weight of nude mice were measured. (e) HE staining showed that maslinic acid significantly inhibited the growth of tumor cells. (f) Immunohistochemical staining showed that maslinic acid significantly inhibited the expression of the proliferation protein PCNA of tumor cells. ^*∗∗∗∗*^*p* < 0.0001. Scale bar = 100 *μ*m.

To verify whether maslinic acid could inhibit the growth of glioma cells in vivo, control solvent (0.9% physiological saline) and the appropriate maslinic acid (20 mg/kg·d) were subcutaneously injected into the model mice subjected to the injection of U251 cells. Tumor pictures and nude mice pictures are shown in Figures 7(a) and 7(d), respectively. As expected, there were statistically significant differences in tumor volume (Figure 7(b)) and weight (Figure 7(c)) between the experimental group and the control group. Simultaneously, histopathological analysis (Figures 7(e) and 7(f)) also showed that the appropriate doses of maslinic acid could lead to the reduction of proliferation of the glioma cells in vivo.

### 3.6. Discussion

In the past few decades, plant-derived drugs have received more and more attention because they are relatively nontoxic and cheap compared to modern drugs [18]. Medicinal plants and their extracts are widely used because of their antiinflammatory, antioxidant, antidiabetic, anticancer, and other biological properties [19–21]. A large number of studies have shown that the extracts of many medicinal plants are good for anticancer, such as paclitaxel, camptothecin, and vincristine, and their analogs have been widely used clinically [22]. Maslinic acid is an effective ingredient extracted from olives and belongs to a kind of triterpenoids. Many studies have shown that maslinic acid has anti-inflammatory, antitumor, antidiabetic, antioxidant, and other biological activities [23]. The anticancer activity of maslinic acid has been reported in a variety of cancers, but few studies have explored its efficacy in gliomas. Despite recent advances in imaging diagnosis, surgical treatment, and radiotherapy, the prognosis of malignant glioma is still very poor [24]. It is urgent to find new chemotherapy drugs to improve the quality of life and prognosis of patients.

In this study, we explored the effects of maslinic acid on the proliferation, apoptosis, migration, and invasion, and tumor growth of glioma cell lines U87 and U251. Our data show that maslinic acid has a concentration-dependent and time-dependent cytotoxic effect on human glioma cells in vitro (Figure 1(b)). In addition, with the increase in the dose of maslinic acid, it has a significant inhibitory effect on the migration and invasion of glioma cells (Figures 4 and 5). In addition, maslinc acid induces glioma cell apoptosis through mitochondria and death receptor pathways (Figure 6). In the nude mice model, maslinic acid can inhibit the growth of glioma and inhibit the expression of the proliferation protein PCNA (Figure 7(f)). Our findings provide a reasonable basis for maslinic acid as a potential complementary treatment for glioma.

MAPK regulates various cell activities related to cancer development, including proliferation, differentiation, apoptosis, inflammation, and immunity [25]. A large amount of evidence has proved that MAPK is a key pathway and molecular target to control tumor progression and survival in a variety of cancers including pancreatic cancer, breast cancer, and liver cancer [26]. MAPK signaling cascades are composed of at least three hierarchically sequential kinase components: a MAPK kinase (MAP3K), a MAPK kinase (MAP2K), and a MAPK. MAP3Ks phosphorylate and activate MAP2Ks, which in turn phosphorylate and activate MAPKs [25]. Activated MAPKs phosphorylate diverse target proteins including transcription factors such as c-Jun, c-Myc, and ATF2 as well as antiapoptotic and proapoptotic proteins such as Bcl-2 and Bax, respectively [27]. At present, the combination of RNA-Seq technology and bioinformatics analysis can explore the abnormal expression of mRNA during the development of gliomas. In this study, through RNA-Seq analysis of the glioma control group and the maslinic acid treatment group, the gene expression in the different treatment groups was obtained. After maslinic acid treatment, KEGG enrichment analysis showed that the MAPK signaling pathway was significantly changed. Among the MAPK signaling pathway-related genes, 29 genes were downregulated (Figure 6(b)) and 13 genes were upregulated (Figure 6(c)). Among them, MAP2K3 was upregulated, and MAP3K1, MAP2K6, and MAPK10 were downregulated. Western blotting results showed that maslinic acid upregulated the expression of p-ERK. Inhibitors targeting the MAPK pathway have shown promising clinical responses in patients with melanoma, non-small-cell lung cancer, colorectal cancer, pancreatic cancer, and thyroid cancer [28]. Consistent with these findings, our results suggest that maslinic acid can inhibit the malignant progression of glioma cells by inhibiting the MAPK pathway. More interestingly, the action of maslinic acid significantly changed the expression of 19 apoptosis-related genes (Figure 6(d)). Bcl-2 and its family members are essential to the intrinsic apoptotic pathway, which involves the mitochondria. Bcl-2, together with Bax, alters the mitochondrial membrane potential and permeability, which initiates the release of regulatory proteins that activate caspases [29]. Furthermore, we performed Western blotting experiments to verify the sequencing results. The results showed that maslinic acid upregulated the expression of proapoptotic family members (caspase-3 and Bax) but decreased the expression of the antiapoptotic protein BCL-2 (Figure 6(a)). This is consistent with the sequencing results. The study of Teng Guan used maslinic acid for the treatment of cerebral ischemic injury in hyperglycemic rats, given intragastrically, and found that maslinic acid had the effect of reducing cerebral ischemic damage in hyperglycemic rats [30], suggesting that maslinic acid may play a key role through the blood-brain barrier. In recent years, the cross-development of the blood delivery system-brain barrier (BBB) and blood-brain tumor barrier (BBTB) provide a new treatment scheme for glioma and malignant brain metastasis. Peptide carrier drug conjugate (PDC), which recognizes the penetration of BBB/BBTB, has been derived and applied to the treatment of glioma metastasis. When combined with the currently used chemotherapeutic drug temozolomide, synergistic antitumor activity was achieved in the tumor model. The peptide can be used as a general carrier to shuttle compounds to BBB [31]. Therefore, as the next step, we will continue to study these models to help maslinic acid pass through the blood-brain barrier.

## 4. Conclusion

In summary, this study confirmed that maslinic acid has an inhibitory effect on the malignant phenotype of glioma cell lines U87 and U251. The first high-throughput method to prove that the inhibitory effect of maslinic acid on the growth of human glioma cells may be achieved by the activation of apoptosis induction through MAPK and caspase/Bcl2/Bax signaling pathways.

## Data Availability

The original contributions presented in the study are included in the article/Supplementary Material. Further inquiries can be directed to the corresponding author.

## Supplementary Materials

Supplementary Materials Supplementary Figure S1: maslinic acid of 30 *μ*m does not affect the proliferation of HK-2, HPDE, and HUVEC cells. Data were representative of three independent experiments, expressed as mean ± SD.
