# Supplementary material for: Maslinic Acid Inhibits the Growth of Malignant Gliomas by Inducing Apoptosis via MAPK Signaling
Source: J Oncol. 2022 Jun 28;2022:3347235. doi: 10.1155/2022/3347235 (PMC9256398; doi:10.1155/2022/3347235)
Supplement: Supplementary Materials — Supplementary Figure S1: maslinic acid of 30 μm does not affect the proliferation of HK-2, HPDE, and HUVEC cells. Data were representative of three independent experiments, expressed as mean ± SD. [file 3347235.f1.zip › 3347235.f1/FIGURES1.pptx]

## Slide 1
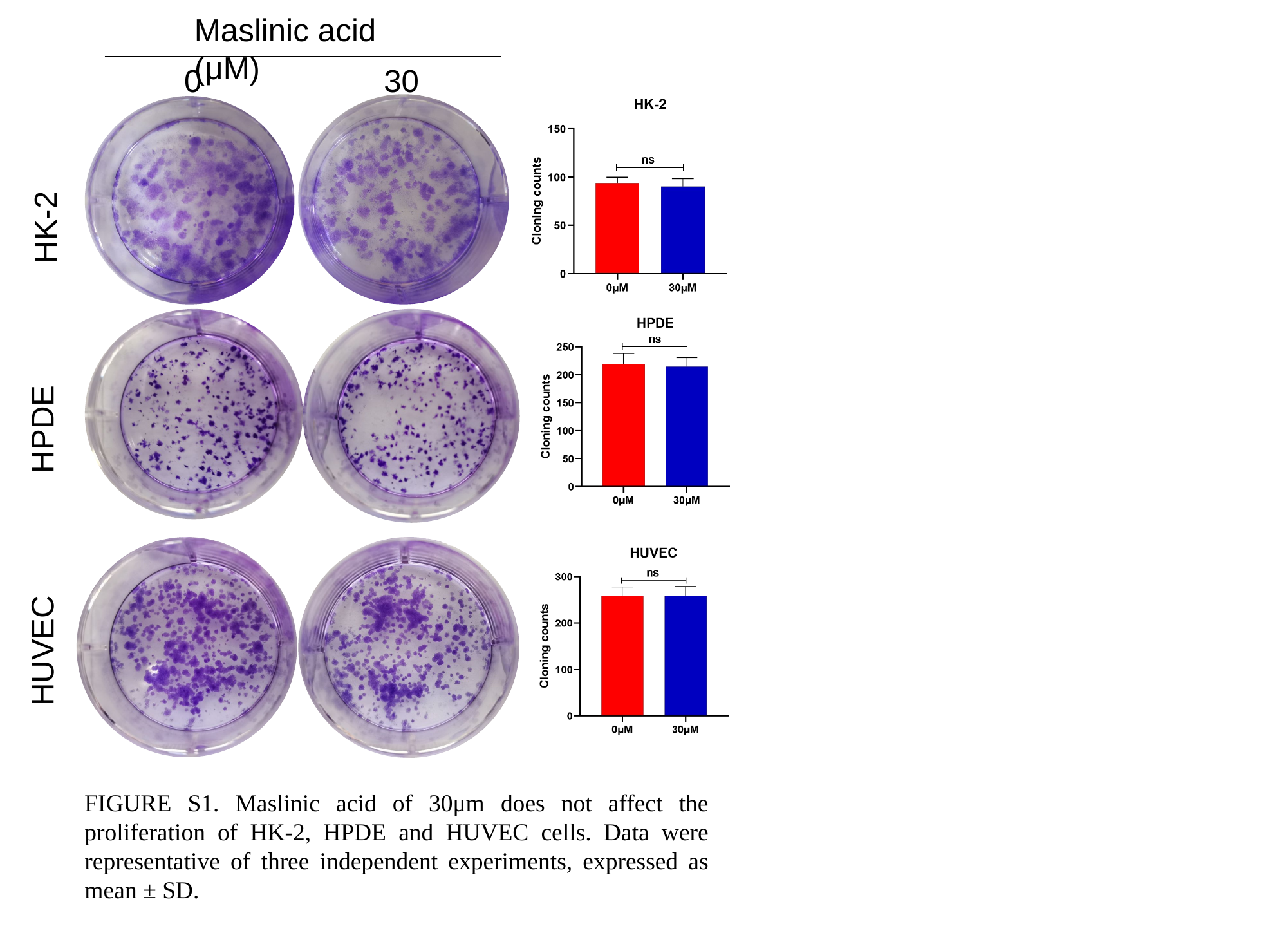

Maslinic acid (μM)
0
30
HK-2
HPDE
HUVEC
FIGURE S1. Maslinic acid of 30μm does not affect the proliferation of HK-2, HPDE and HUVEC cells. Data were representative of three independent experiments, expressed as mean ± SD.
